# Uterine prolapse in pregnancy

**DOI:** 10.11604/pamj.2015.22.188.8077

**Published:** 2015-10-23

**Authors:** Mohamed Amine Ben Haj Hassine, Haythem Siala

**Affiliations:** 1 Service de Gynecologie, Hôpital Militaire de Tunis, Mont FLeury 1008 Tunis, Tunisie

**Keywords:** Pregnancy, prolapse, delivery

## Image in medicine

A 32 year old woman gravida 3 para 2 was admitted in her 33 ^rd^ week of pregnancy for management of uterine prolapse in pregnancy. Her first child was age of 3 years and was born by forceps assisted vaginal delivery, weighting 3,8 kg. She had no history of uterine prolapse prior to the current pregnancy. In the 12 ^th^ week of gestation, she noticed a protrusion from her vagina. She had no other symptoms. She was able to reduce the protrusion regularly. In the 33 ^rd^ week of gestation, she presented with uterine contractions. The os of the prolapsed cervix was closed and elongated. Her contractions were successfully inhibited with calcic inhibitors. At 37 weeks of gestation, a viable male infant weighting 2,6 kg was delivered vaginally following rupture of membranes. She progressed well in labour. The placenta was delivered spontaneously. a vaginal ring pessary was inserted two days later to control the prolapse during the period of involution. After 6 months, she had no longer uterine prolapse.

**Figure 1:**
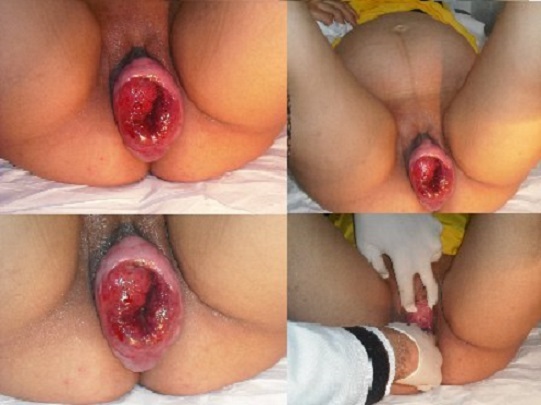
Uterine prolapsed in pregnancy

